# Human CD8^+^ EMRA T cells display a senescence‐associated secretory phenotype regulated by p38 MAPK

**DOI:** 10.1111/acel.12675

**Published:** 2017-10-12

**Authors:** Lauren A. Callender, Elizabeth C. Carroll, Robert W. J. Beal, Emma S. Chambers, Sussan Nourshargh, Arne N. Akbar, Sian M. Henson

**Affiliations:** ^1^ Translational Medicine and Therapeutics, William Harvey Research Institute Barts and The London School of Medicine and Dentistry Queen Mary University of London Charterhouse Square London EC1M 6BQ UK; ^2^ Microvascular Research, William Harvey Research Institute Barts and The London School of Medicine and Dentistry Queen Mary University of London Charterhouse Square London EC1M 6BQ UK; ^3^ Division of Infection and Immunity University College London London WC1E 6JF UK

**Keywords:** aging, cytokine, inflammation, microarray, SASP, T cell

## Abstract

Cellular senescence is accompanied by a senescence‐associated secretory phenotype (SASP). We show here that primary human senescent CD8^+^ T cells also display a SASP comprising chemokines, cytokines and extracellular matrix remodelling proteases that are unique to this subset and contribute to age‐associated inflammation. We found the CD8^+^ CD45RA ^+^ CD27^−^ EMRA subset to be the most heterogeneous, with a population aligning with the naïve T cells and another with a closer association to the effector memory subset. However, despite the differing processes that give rise to these senescent CD8^+^ T cells once generated, they both adopt a unique secretory profile with no commonality to any other subset, aligning more closely with senescence than quiescence. Furthermore, we also show that the SASP observed in senescent CD8^+^ T cells is governed by p38 MAPK signalling.

## Introduction

Immune senescence results from defects in T‐cell immunity and is also characterized by a low‐grade chronic inflammatory state (Macaulay *et al*., 2013). Little is known about the source of the inflammation that fuels most age‐related diseases; however, it may derive from an age‐related decline in homoeostatic immune function, resistance to endogenous microbes or senescent cells (Tchkonia *et al*., 2013). The senescent phenotype is not just proliferative arrest; rather, it is a widespread change in protein expression and secretion, including pro‐inflammatory cytokines, chemokines, growth factors and proteases, termed the senescence‐associated secretory phenotype or SASP (Coppe *et al*., 2008). Consequently, senescent cells can alter the tissue microenvironment and affect neighbouring cells through paracrine signalling. The SASP is highly conserved between species (Coppe *et al*., 2010) and occurs in a variety of cells types (Erusalimsky & Kurz, 2005; Salminen *et al*., 2011) that may be specifically adapted to control different biological processes (Akbar *et al*., 2016).

The SASP was originally thought to result from persistent activation of the DNA damage response (Rodier *et al*., 2009); however, it is now known to be regulated by p38 MAPK, which was shown to be both necessary and sufficient for its development in fibroblasts (Freund *et al*., 2011). The chronic and sustained activation of p38 MAPK differs substantially from the response to acute stress and was found to follow the kinetics of SASP development. Furthermore, siRNA interference of p38 MAPK was shown to significantly reduce the secreted levels of most SASP factors (Freund *et al*., 2011). To date, the SASP has predominantly been characterized in fibroblast cell culture models or aged mice (Coppe *et al*., 2008, 2010; Aoshiba *et al*., 2013), with very few reports of a SASP being found in the human immune system with either age or differentiation (Frasca *et al*., 2017).

Senescent CD8^+^ T cells are found within the CD27^−^CD28^−^ population (Weng *et al*., 2009; Parish *et al*., 2010), and these highly differentiated T cells can be further divided using CD45RA. T cells that re‐express CD45RA within this subset have multiple characteristics of senescence, including a low proliferative activity, high levels of DNA damage and the loss of telomerase activity (Henson *et al*., 2014, 2015). We have also shown that p38 MAPK signalling, which is increased in highly differentiated CD8^+^ T cells (Henson *et al*., 2014), is involved in the loss of telomerase activity and proliferative capacity and that blockade of p38 MAPK activity with a specific small‐molecule inhibitor can restore both proliferation and telomerase activity (Lanna *et al*., 2013; Henson *et al*., 2014, 2015) in these cells. However, surprisingly the CD45RA‐re‐expressing senescent T cells do not have critically short telomeres (Di Mitri *et al*., 2011; Riddell *et al*., 2014), suggesting that senescence in these cells may be induced by other mechanisms including DNA damage by increased ROS production (Henson *et al*., 2014). Indeed, we show here that the CD45RA^+^CD27^−^ T cells are the most heterogeneous CD8^+^ T‐cell subset that pertain from either the naïve or effector memory CD8^+^ T cells, and carry with them hallmarks of each of these subsets.

In this study, we demonstrate that irrespective of the derivation of CD8^+^ CD45RA^+^CD27^−^ T cells, these primed cells exhibit a unique highly inflammatory secretory profile characteristic of the SASP, and we also provide evidence that ADAM28 can be used as a functional marker of senescence in CD8^+^ T cells. Furthermore, we show that the secretory phenotype in CD8^+^ CD45RA^+^CD27^−^ T cells is controlled through p38 MAPK signalling, which contributes to age‐associated inflammation.

## Results

### CD8^+^ T cells display a senescence‐associated secretory phenotype

**Figure 1:**
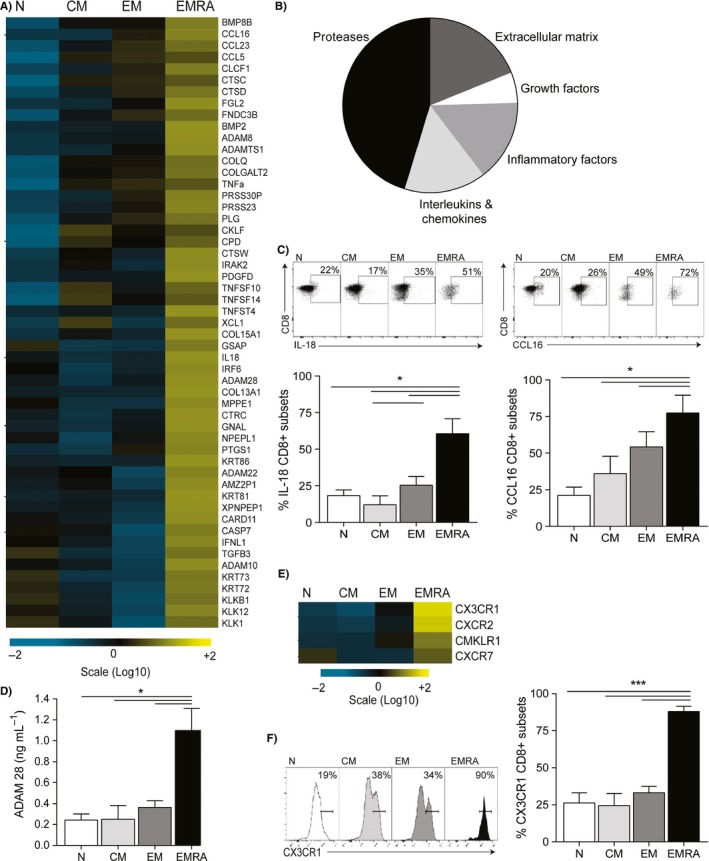
CD8^+^ EMRA T cells display a unique secretory phenotype. (A) Shown are the secreted factors which were significantly increased (*P *< 0.05) in the CD8+ EMRA T‐cell subset. For each protein, the relative gene expression is displayed as the average value of six donors. Signals above baseline are yellow; signals below baseline are blue. The heat map key shows log‐fold changes from baseline. (B) Pie chart showing the relative percentage of secreted factors split into key functions. (C) Representative flow cytometry plots and cumulative data of IL‐18 and CCL16 on CD8^+^ CD45RA/CD27‐defined T‐cell subsets following an 18‐hour stimulation with 0.5 μg mL^−1^ anti‐CD3. Graphs show the mean ± SEM for four donors. (D) Graph showing the production of ADAM28 by the four CD8^+^ T‐cell subsets following an 18‐hour stimulation with 0.5 μg mL^−1^ anti‐CD3 and 5 ng mL^−1^ IL‐2. Graph shows the mean ± SEM for three donors. (E) Heat map showing the relative gene expression of chemokine receptors that were significantly upregulated (*P *< 0.05) by the CD8^+^ EMRA T‐cell subset. (F) Flow cytometry plot and graph showing the expression of CX3CR1 on the four CD45RA/CD27‐defined CD8^+^ T‐cell subsets. Graph shows the mean ± SEM for four donors. *P*‐values were calculated using a repeated‐measures ANOVA with the Tukey correction used for post hoc testing.

To determine whether senescent CD8^+^ T cells exhibit a secretory phenotype similar to that seen in senescent fibroblasts (Rodier *et al*., 2009), we compared the gene expression profiles of CD45RA/CD27‐defined CD8^+^ T‐cell subsets using Affymetrix U133 plus 2 arrays. The gene expression patterns of sorted central memory (CM; CD45RA^‐^CD27^+^), effector memory (EM; CD45RA^−^CD27^−^) and effector memory T cells that re‐express CD45RA (EMRA; CD45RA^+^CD27^−^) CD8^+^ T cells were compared to the naïve (N; CD45RA^+^CD27^+^) CD8^+^ subset so that direct comparisons between all three groups could be made. Six individuals were chosen for their even distribution of CD8^+^ T cells between the four CD45RA/CD27‐defined subsets: average N, 38%; CM, 21%; EM, 7%; and EMRA, 25%. We found that 1472 genes were differentially expressed only in the senescent EMRA population when compare to the N subset (FDR‐corrected *P*‐value higher than or equal to 0.01). The number of unique genes fell to 519 when the EMRA population was compared to the EM cells, indicating that EMRA CD8^+^ T cells reflect a unique state of gene expression. When analysed for genes relating to soluble factors secreted by the senescent EMRA CD8^+^ T cells, we found 53 genes to be significantly upregulated (Fig. 1A). The SASP components in CD8^+^ EMRA T cells are comprised mainly of proteases, chemokines and interleukins as well as other inflammatory, growth and insoluble factors such as extracellular matrix components (Fig. 1B). Of the inflammatory and immune‐modulatory cytokines and chemokines, TNF‐α, IL‐18, IL‐29, CCL5, CCL16 and CCL23 were all found to be significantly upregulated in the EMRA subset. We and others have previously published that EMRA T cells secrete high levels of TNF‐α (Hamann *et al*., 1997; Henson *et al*., 2015). However, we validate here the relative increase in gene expression of IL‐18 and CCL16 by flow cytometry, showing them both to be significantly increased in the CD8^+^ EMRA subset (Fig. 1C). Of note, the cytokines IL‐1β and IL‐6, shown to be highly secreted by senescent fibroblasts (Coppe *et al*., 2008), are expressed by the EMRA subset, but the EM cells produce the highest amounts of these cytokines and are not a SASP‐defining feature in CD8^+^ T cells. However, EMRA cells do produce more IL‐6 and IL‐1β than the N or CM subsets (Fig. S1A).

The unique secretory nature of CD8^+^ EMRA T cells also included increased secretion of proteases, 23 of the 53 significantly upregulated secreted factors are proteases (Fig. 1A,B), including cathepsins and serine proteases but also the ADAM family of disintegrin and metalloproteases. In particular, ADAM28, which was found to be one of the most upregulated genes in the EMRA subset, was found to be secreted at significantly higher levels by ELISA in the EMRA subset when compared to the other T‐cell subsets (Fig. 1D). ADAMs are regulatory proteins that pose both proteolytic activity and the ability to modulate intercellular adhesion. Indeed, as well as ADAM28's involvement in membrane‐bound TNF‐α cleavage (Jowett *et al*., 2012), the disintegrin domain of ADAM28 also serves as a ligand for the integrin α4β1 (Bridges *et al*., 2002). Therefore, the upregulation of proteases may also indicate changes to the migratory potential of the EMRA subset. Further evidence for the altered migratory nature of the EMRA CD8^+^ T cells can be found from the unique chemokine receptor profile expressed by these cells (Fig. 1E). With the expression of CX3CR1 directly *ex vivo* on the EMRA subset being >90% by flow cytometry (Fig. 1F), potentially mediating their adhesion to the endothelium in the absence of stimulation. This finding therefore suggests that CD8^+^ EMRA T cells contribute to local inflammation and the amplification of the inflammatory response owing to their adhesion to the endothelium and the recruitment of other inflammatory mediators.

### Regulation of the secretory phenotype seen in CD8^+^ CD45RA^+^CD27^−^ T cells

**Figure 2:**
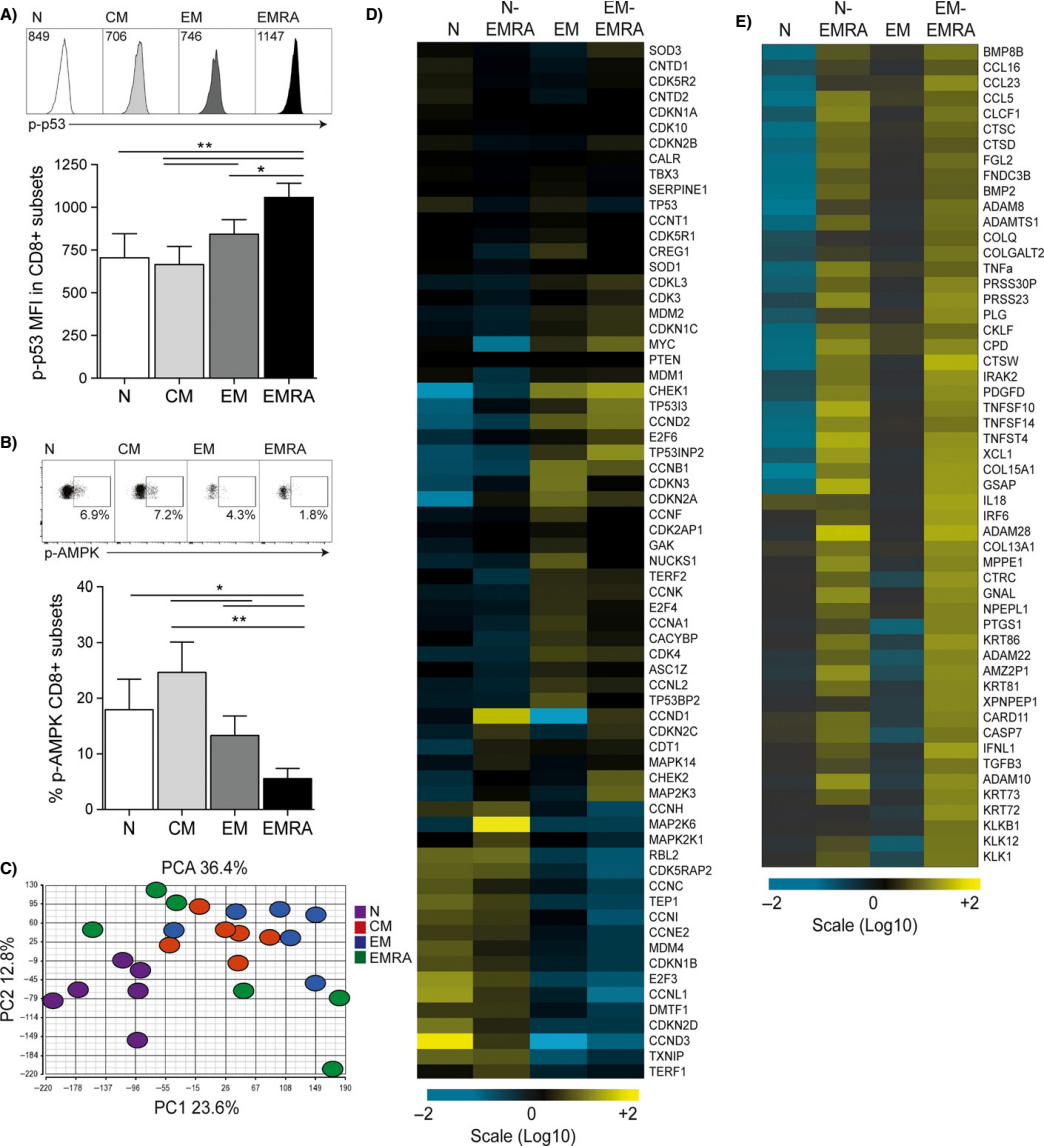
Regulation of the secretory phenotype seen in CD8^+^ EMRA T cells. (A) Representative flow cytometry plots of phosphorylated p53. Graph depicts the expression of p‐p53 in CD8^+^ CD45RA/CD27‐defined T‐cell subsets. Graph shows the mean ± SEM for nine donors. (B) Flow cytometry plots and graph showing the expression of phosphorylated AMPK in CD8^+^ T‐cell subsets following an 18‐hour stimulation with 0.5 μg mL^−1^ anti‐CD3. Graph shows the mean ± SEM for 10 donors. *P*‐values were calculated using a repeated‐measures ANOVA with the Tukey correction used for post hoc testing. (C) PCA of CD8^+^ CD45RA/CD27‐defined T‐cell subsets. (D) Heat map showing the relative gene expression of senescence genes for the N and EM CD45RA/CD27‐defined subsets (six donors) compared to the naïve‐like and EM‐like EMRA subsets (three donors each). (E) Relative gene expression changes for genes controlling the secreted SASP factors in N and EM CD45RA/CD27‐defined subsets compared to the naïve‐like and EM‐like EMRA subsets. The heat map keys show log‐fold changes from baseline.

It has recently been shown that dysfunctional mitochondria can promote a distinct secretory phenotype in human cells and mice termed mitochondrial dysfunction‐associated senescence (MiDAS) (Wiley Christopher *et al*., 2016). The authors showed that the MiDAS resulted from an NADH‐AMPK‐p53‐dependent pathway that elicited a SASP but lacked IL‐1‐dependent factors. We have previously shown CD8^+^ EMRA T cells to display mitochondrial dysfunction (Henson *et al*., 2014). Therefore, we investigated whether the CD8^+^ EMRA SASP was also governed by a p53‐AMPK‐dependent pathway. However, while we found the EMRAs to express the highest level of p‐p53 (Fig. 2A), they, unlike their CD4^+^ senescent counterparts (Lanna *et al*., 2014), did not display any AMPK phosphorylation, either directly *ex vivo* (Fig. S1B) or following stimulation (Fig. 2B). Why this should be the case is unclear, but it may be due to the differing roles of CD4^+^ and CD8^+^ T cells; furthermore, CD4^+^ T cells have been shown to have a higher mitochondrial mass than CD8^+^ T cells (Cao *et al*., 2014) regulated by AMPK (Jornayvaz & Shulman, 2010).

We found that the CD45RA^+^CD27^−^ EMRA subset was the most heterogeneous of the four subsets, shown here by PCA (Fig. 2C), with a population aligning with the naïve subset and a population with a closer association with the effector memory subset. Furthermore, unsupervised clustering revealed the EMRA populations to align with the EM and N populations (data not shown). We then split the EMRA subset into two groups depending on whether they aligned to the naïve subset, naïve‐like EMRAs, effectors or effector‐like EMRAs and analysed the microarray data for genes relating to senescence and the SASP (Fig. 2D and E, respectively). Indeed, when comparing the gene expression profiles of the naïve and naïve‐like EMRAs and EM and EM‐like EMRAs for genes associated with senescence, we found the naïve subsets to be closely aligned, as are the EM subsets (Fig. 2D). Each EMRA subset expressed cell cycle inhibitors more closely allied to its cognate counterpart; naïve and naïve‐like EMRAs displayed higher expression levels of cyclin D (ccnd1), pRB and erk (map2k6), while the EM and EM‐like EMRAs showed increased amounts of Chk1 (chek1) and TP53I3. These cell cycle regulators and kinases can switch on senescence or quiescence phenotypes; however, genes controlling telomerase activity such as TERT and the shelterin complex are repressed in both EMRA populations, suggesting that both these EMRA phenotypes are senescent rather than quiescent (Fig. S1C). In addition, when assessing genes associated with the SASP (Fig. 2E), we found no alignment of the two EMRA populations with either the naïve or the EM subsets, indicating that despite the differing processes that give rise to these senescent CD8^+^ T cells once generated, they both adopt a unique secretory profile with no commonality to any other subset, aligning more closely with senescence than quiescence.

**Figure 3:**
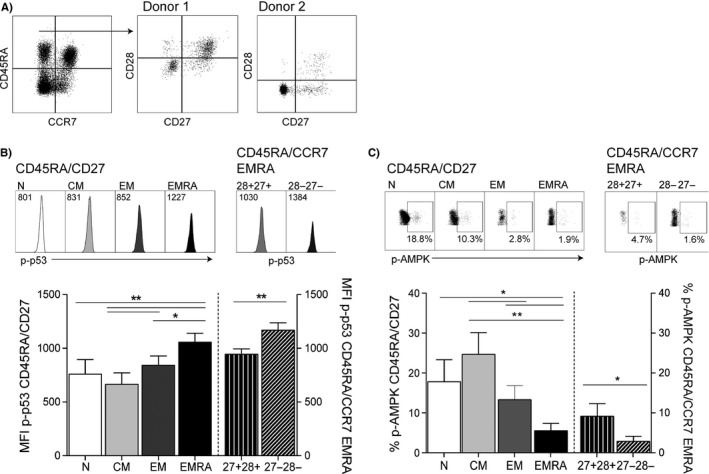
Characterization of the senescent profile of CD8^+^ CD45RA ^+^ CCR7^−^ CD28/CD27 T cells. (A) Representative flow cytometry plots showing phenotypic staining using CD45RA/CCR7 and the different CD28/CD27 staining pattern observed in CD45RA/CCR7‐defined EMRA cells from two donors. (B) Flow cytometry plots and graphs depicting p‐p53 expression in ex vivo CD8^+^ CD45RA/CD27‐defined (left axis) and CD45RA ^+^ CCR7^−^ CD28/CD27‐defined subsets (right axis). (C) Plots and graph showing the expression of phosphorylated AMPK in CD8^+^ T‐cell subsets following an 18‐h stimulation with 0.5 μg mL^−1^ anti‐CD3 in the above subsets. Graphs show the mean ± SEM for nine donors. *P*‐values were calculated using a paired t‐test or a repeated‐measures ANOVA with the Tukey correction used for post hoc testing.

Analysis of the microarray data revealed that a key difference between the two EMRA populations was the expression of CD28, with N‐like EMRAs showing high expression levels of this molecule (Fig. S1D). Therefore, to formally investigate the heterogeneity of the EMRA subset, we used CCR7, CD45RA, CD28 and CD27 to define senescence. When CD8^+^ T cells are gated on CD45RA^+^CCR7^−^ cells, this highly differentiated population is found to be highly heterogeneous. This population was composed of a naïve‐like population expressing both CD27 and CD28 and more differentiated population that have lost both these markers (Fig. 3A). The CD28^+^CD27^+^ and CD28^−^CD27^−^ population were found to differentially express p‐p53; however, both populations expressed higher levels of p‐p53 than all other CD8^+^ T‐cell subsets (Fig. 3B). When we assessed the expression of p‐AMPK in the CD28^+^CD27^+^ and CD28^−^CD27^−^ senescent populations, we found the CD28^+^CD27^+^ population to express high levels of p‐AMPK, akin to the expression levels observed in less differentiated CD45RA/CD27 T‐cell subsets (Fig. 3C), indicating that there is a naïve‐like population of senescent CD8^+^ T cells displaying a unique senescent phenotype expressing p53 and AMPK and an EM‐like population expressing p53 only.

### MAPK activity is necessary to induce a SASP in senescent CD8^+^ T cells

**Figure 4:**
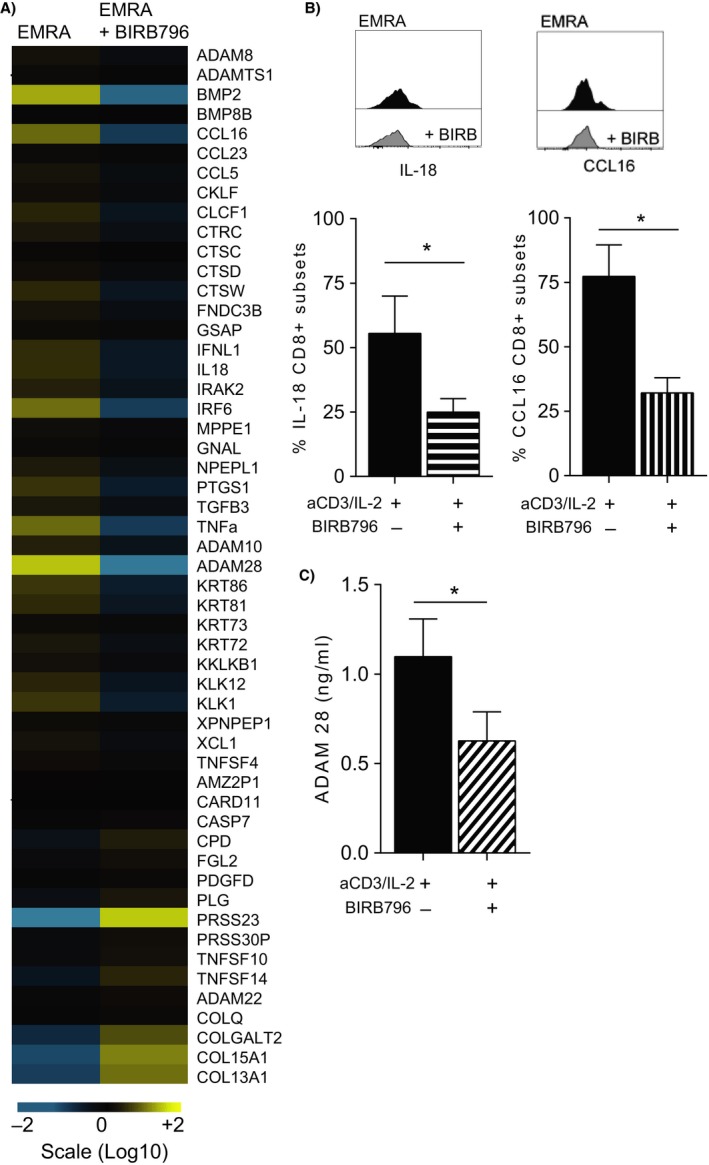
The secretory phenotype of CD8^+^ EMRA T cells is regulated in a p38 MAPK‐dependent manner. (A) Heat map showing the changes in the secreted factors produced by CD8^+^ EMRA T cells following inhibition of the p38 MAPK pathway using BIRB796. For each protein, the relative gene expression is displayed as the average value of six donors. Signals above baseline are yellow; signals below baseline are blue. The heat map key shows log‐fold changes from baseline. (B) Flow cytometry plots and cumulative data of IL‐18 and CCL16 in CD8^+^ EMRA T‐cell subset following an 18‐hour stimulation with or with our BIRB796. Graphs show the mean ± SEM for four donors. (C) Graph showing the production of ADAM28 from stimulated CD8^+^ EMRAs following blockade of the p38 MAPK pathway using BIRB796. Graph shows the mean ± SEM for three donors. *P*‐values were calculated a repeated‐measures ANOVA with the Tukey correction used for post hoc testing.

We then went on to investigate whether the SASP observed by the naïve‐ or EM‐like EMRA subset was regulated in a p38 MAPK‐dependent manner, like that observed in fibroblasts (Freund *et al*., 2011). Likewise, we show here that the CD8^+^ EMRA T‐cell SASP is also controlled by p38 MAPK, for blocking the p38 MAPK pathway with the small‐molecule inhibitor BIRB 796 resulting in the downregulation of 34 of the 53 secreted factors (Fig. 4A). We found the relative gene expression profiles of all the cytokines and chemokines to be reduced regardless of EMRA subset, with the responses of IL‐18 and CCL16 in the four CD27/CD45RA‐defined subsets being validated by flow cytometry (Fig. 4B). In addition, the expression of over half the secreted proteases was also reduced, shown here by the decline in ADAM28 production measured by ELISA (Fig. 4D). Finally, while the production of IL‐1β and IL‐6 was not highly expressed in the CD8^+^ EMRA subset, their production was controlled by p38 MAPK (Fig. S1E). In summary, we show here that the senescent CD8^+^ EMRA population has a unique secretory phenotype governed by p38 MAPK.

## Discussion

Senescence was initially described as an irreversible cell cycle arrest; however, it has now been shown to be more than a process of reduced proliferative capacity but rather an active process regulating cellular homoeostasis in response to numerous stresses, such as DNA damage or a reduction in growth factors (Campisi, 2005; Akbar & Henson, 2011). The ability of senescent cells to modulate their growth‐promoting pathways marks them as different from quiescent cells which are held in a nonproliferating state. The hyperfunctional nature of senescent cells is characterized by the secretion of growth factors, inflammatory mediators, proteases, extracellular matrix components termed the senescence‐associated secretory phenotype (SASP). These secreted factors accumulated in tissues and altered the structure and cellular microenvironment (Campisi *et al*., 2011). We show here that senescent CD8^+^ CD45RA/CD27‐defined EMRA T cells display a unique secretory profile characteristic of the SASP.

The components of the SASP have been shown to vary depending on the cell type, with differing levels of cytokines and other inflammatory mediators being found in epithelial cells and mesenchymal cells (Xu *et al*., 2015). Indeed, we show here that senescent CD8^+^ T cells exhibited a SASP which differs to that reported for fibroblasts and B and NK cells, in that it is not defined by the expression of IL‐1β and IL‐6. This may relate to our finding that EMRA T cells are unable to phosphorylate mTOR (Henson *et al*., 2014), a key molecule in the stabilization and translation of inflammatory cytokine mRNAs (Kafasla *et al*., 2014). However, senescent CD8^+^ T cells do secrete high levels of IL‐18, a pro‐inflammatory cytokine in the IL‐1 family that induces IFN‐γ production. Nevertheless, CD8^+^ EMRA T cells do produce more inflammatory cytokines than EM T cells, potentially reinforcing both the senescent and inflammatory phenotype of these cells.

We also demonstrate the CD8^+^ T‐cell SASP to predominantly comprise proteases, which have the potential to alter tissue structure and organization through the cleavage of membrane‐bound receptors, signalling ligands, extracellular matrix proteins or other components in the tissue microenvironment (van Deursen, 2014). One protease in particular, ADAM28, was found to be highly upregulated in senescent CD8^+^ T cells and offers a novel functional biomarker of T‐cell senescence. ADAMs (a disintegrin and metalloprotease) are a family of transmembrane proteins which control interactions with the extracellular matrix through proteolytic modification of cell surface proteins, as well as acting as adhesion molecules (Seals & Courtneidge, 2003). ADAM28 has been shown to be involved in membrane‐bound TNF‐α cleavage (Jowett *et al*., 2012), as well as serving as a ligand for the integrin α4β1, where it is thought to target the active protease to substrates at the site of cell–cell contact (Bridges *et al*., 2002). Additionally, ADAM28 has also been shown to bind P‐selectin glycoprotein ligand‐1 (PSGL‐1) enhancing cell adhesion to endothelial cells and subsequent migration into tissues (Shimoda *et al*., 2007). Therefore, the enhanced expression of ADAM28 in senescent CD8^+^ T cells together with high expression levels of CX3CR1 directly ex vivo has the potential to alter the migration of these cells. The increased inflammatory nature of senescent CD8^+^ T cells is associated with acute renal rejection and vascular injury (Dedeoglu *et al*., 2016); furthermore, the adhesion and migration of T cells in inflamed vessels have also been linked to endothelial barrier dysfunction (Yang *et al*., 2009). Therefore, it will be instructive to assess the damage senescent T cell cause by their extravasation.

The CD8^+^ CD45RA/CD27‐defined EMRA subset was also found to be highly heterogenic, displaying characteristics of either naïve or effector memory cells. Heterogeneity within memory T cells has been documented to occur during aging (Henson *et al*., 2015; Pulko *et al*., 2016). A human memory population with a naïve‐like phenotype (CCR7^+^CD45RA^+^CD28^int^CD95^lo^) was found to increase during aging and exhibited a transcriptome distinct from other T‐cell subsets (Pulko *et al*., 2016). The authors found this subset to have long telomeres and respond to persistent viral infections. We show here the diverse nature of a more differentiated subset of memory cells and postulate that unlike the naïve memory cells, these EMRA cells are an antigen‐experienced functionally senescent population with characteristics of naïve or effector cells generated via different mechanisms. Senescence can be triggered via many routes, and as such, the SASP components also vary; for example, primary lung fibroblasts generated via proteasome inhibition showed a unique combination of SASP constituents compared to the induction of senescence through replication or oxidative stress (Maciel‐Barón *et al*., 2016). Ongoing work suggests that the senescent phenotype of our EMRA subset is generated by replicative senescence and homoeostatic mechanisms, but the resulting SASP, while not identical, represents a unique phenotype that is distinct from the other T‐cell subsets.

We also demonstrated that the senescent CD8^+^ EMRA T‐cell SASP is predominantly but not totally governed by p38 MAPK signalling, akin to that demonstrated for fibroblasts (Freund *et al*., 2011) and B cells (Frasca *et al*., 2017). In these fibroblast and B‐cell models, p38 MAPK activated a SASP downstream of AMPK, and we show here that in the naïve‐like EMRA subset, this may also be the case; however, the EM‐like EMRAs were found to be governed independently of AMPK. The existence of AMPK‐independent mechanisms has been demonstrated; for example, the induction of senescence by genotoxic stress gives rise to a secretory phenotype regulated by NF‐ κB and not AMPK (Wiley Christopher *et al*., 2016). We show here a population of naïve‐like senescent cells that produced a secretome potentially via AMPK and an EM‐like population that lack AMPK activity; therefore, a fuller analysis of the controlling factors in the EMRA subsets is warranted.

In summary, despite the plasticity of the CD8^+^ EMRA T‐cell subset, the resulting cells are functionally senescent, as demonstrated by both proliferative arrest and the increased production and secretion of inflammatory mediators characteristic of a SASP. The components of this secretory phenotype appear to be tailored to the unique migratory behaviour of the EMRA T cell with the potential to enhance their pathogenicity.

## Experimental procedures

### Blood sample collection and isolation

Heparinized peripheral blood samples were taken from healthy volunteers (age range: 32–55 *n *= 6). Further, donors were recruited to validate observations arising from microarray analysis (age range: 24–45). Healthy volunteers were individuals who had not had an infection or immunization within the last month, no known immunodeficiency or any history of chemotherapy or radiotherapy, and were not receiving systemic steroids within the last month or any other immunosuppressive medications within the last 6 months. PBMC were isolated using Ficoll‐Paque (Amersham Biosciences, Little Chalfont, Buckinghamshire, UK). All samples were obtained in accordance with the ethical committee of Royal Free and University College Medical School and the North East‐York Research Ethics Committee 16/NE/0073.

### Flow cytometric analysis and cell sorting

Flow cytometric analysis was performed using the following antibodies: CD8 PerCP (SK1), CD45RA BV605 (HI100), CD27 BV421 (O323), CD28 BV785 (CD28.2), CCR7 PE‐Cy7 (G0343H7), KLRG1 PE (2f1/KLRG1) and CX3CR1 (K0I24E1), from BioLegend. For intracellular staining, the following antibodies were used: IL‐18/IL‐1F4 propeptide PE (74801) and CCL16/HCC‐4 FITC (70218) (both from R&D Systems); Ki67 PE (Ki67), p53 Alexa Fluor 647 (1C12), p‐p53 (Ser15, 16G8) Alexa Fluor 647 and p‐AMPK (Thr172, 40H9) (all from Cell Signaling Technology); and goat anti‐rabbit IgG H&L Alexa Fluor 488 (Abcam, Milton, Cambridge, UK). Detection of IL‐18, CCL16 and p53 was carried out following an 18‐hour stimulation with plate‐coated anti‐CD3 (OKT3), and p‐AMPK was detected after a 2‐hour starvation period in PBS followed by a 1‐hour stimulation with plate‐bound anti‐CD3 (OKT3). Following surface staining, PBMCs were fixed in solution A (ThermoFisher, Paisley, Renfrewshire, UK) for 15 min at room temperature followed by permeabilization with solution B (ThermoFisher, Paisley, Renfrewshire) plus antibody for 20 min at room temperature. All samples were run using an LSR II (BD Biosciences, Winnersh, Woking, UK) and analysed using FlowJo software (Treestar).

CD8^+^ T cells were purified by positive selection (Miltenyi Biotec, Bisley, Woking, UK) according to the manufacturer's instructions. Positively selected CD8^+^ T cells were labelled with CD27 FITC (M‐T271) and CD45RA APC (HI100) (both from BD Biosciences) and sorted using a FACSAria (BD Biosciences). The purity of CD8 T‐cell subsets was assessed by flow cytometry.

### Measurement of ADAM28

CD45RA/CD27‐defined CD8^+^ T‐cell subsets were sorted, and 2 × 10^5^ cells were cultured with 0.5 μg mL^−1^ plate‐coated anti‐CD3 (OKT3) and 5 ng mL^−1^ rhIL‐2. Cell lysates were collected at 48 h for the measurement of ADAM28 using the human ADAM28 ELISA kit (LSBio) according to the protocol provided by the manufacturer.

### Cytometric array cytokine profiling

IL‐1β concentration in culture supernatants was measured using the Cytometric Bead Array (BD Biosciences), according to the manufacturer's protocol. The lower limit of detection for each analyte was 1.5 pg mL^−1^.

### p38 MAPK inhibition

Signalling through p38 MAPK on either PBMCs or CD27/CD45RA‐defined CD8^+^ subsets was blocked by adding the small‐molecule inhibitor BIRB796 (Selleck) at a final concentration of 500 nm for the indicated time periods.

### Microarray data acquisition

Cells purified by FACS sorting were stimulated for 2 h with 0.5 μg mL^−1^ plate‐coated anti‐CD3 (OKT3) and 5 ng mL^−1^ rhIL‐2 before RNA isolation using the ARCTURUS PicoPure Isolation Kit (ThermoFisher). The concentration of small quantities of RNA was determined using the Nanodrop. Linear amplification of 10 ng of total RNA was performed using the Ovation Biotin RNA amplification and labelling system (NuGEN). Fragmented, labelled cDNA was hybridized to Affymetrix U133 plus 2 arrays.

### Microarray analysis

Raw microarray intensity data were normalized by robust multi‐array average (RMA) (Irizarry *et al*., 2003). RMA data sets each were then filtered to exclude microRNA, open reading frame, nonprotein coding, pseudogene, antisense, small nucleolar RNA and uncharacterized RNA. Final analysis thus was performed on data sets that contained log‐transformed signal intensities for 18 646 known genes that have a well‐annotated official gene symbol. The analysis of variance (ANOVA) and multitest correction for *P*‐values were used to identify differentially expressed genes. Lists of genes with significant variation of the expression levels (*P *< 0.01) were generated using a 0.02 FDR criterion as a significant cut‐off. Gene Cluster 3.0 software was used to centre the genes and cluster them using Kendall's tau coefficient. Heat maps of the clustered data were created using Java TreeView software (de Hoon *et al*., 2004). Principal component analysis was generated using the Partek software package. The data discussed in this publication have been deposited in NCBI's Gene Expression Omnibus (Edgar *et al*., 2002) and are accessible through GEO Series accession number GSE98640 (https://www.ncbi.nlm.nih.gov/geo/query/acc.cgi?acc=GSE98640).

### Statistical analysis

GraphPad Prism was used to perform statistical analysis. Statistical significance was evaluated using the paired Student *t*‐test or a repeated‐measures ANOVA with the Tukey correction used for post hoc testing. Differences were considered significant when *P* was <0.05.

## Funding

This work was supported by the British Heart Foundation (LAC), a Springboard award from the Academy of Medical Science and the Wellcome trust (ECC, SMH), the William Harvey Research Foundation (SMH), a Wellcome Trust Senior Investigator Award (RWJB, SN) and The Medical Research Council (ESC and ANA) and the British Biotechnology and Biological Research Council (ANA).

## Conflict of Interest

The authors have no conflicting financial interests.

## Author contributions

SMH wrote the manuscript, designed and performed the experiments and analysed the data; LAC, ECC and ESC performed experiments; RWJB analysed the microarray data; SN provided a critique of the manuscript; and ANA designed experiments as well as reviewing the manuscript.

## Supporting information


**Fig. S1** Characteristics of the CD45RA/CD27 defined EMRA population.
