# Supplementary figures and images for: Human CD8+ EMRA T cells display a senescence‐associated secretory phenotype regulated by p38 MAPK
Source: Aging Cell. 2017 Oct 12;17(1):e12675. doi: 10.1111/acel.12675 (PMC5770853; doi:10.1111/acel.12675)

Supplementary Figure 1.

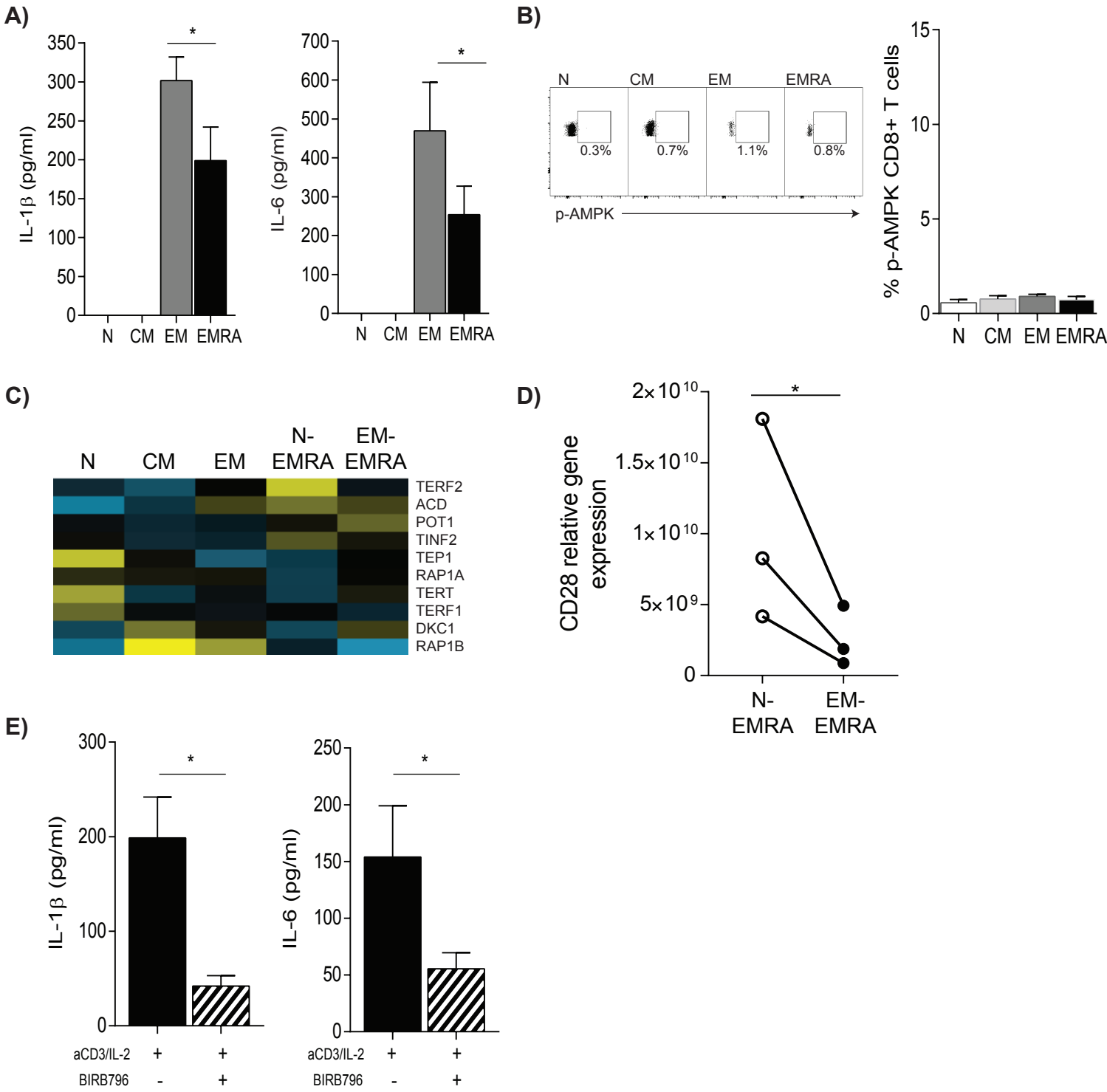

Supplement: Supplementary file 1 — Fig. S1 Characteristics of the CD45RA/CD27 defined EMRA population. [file ACEL-17-na-s001.pdf]
