# Supplementary material for: Human CD8+ EMRA T cells display a senescence‐associated secretory phenotype regulated by p38 MAPK
Source: Aging Cell. 2017 Oct 12;17(1):e12675. doi: 10.1111/acel.12675 (PMC5770853; doi:10.1111/acel.12675)
Supplement: Supplementary file 2 [file ACEL-17-na-s002.docx]

**Supplementary Figures**

**Supplementary Figure 1. Characteristics of the CD45RA/CD27 defined EMRA population.**

(A) Graphs showing the production of IL-1β and IL-6 by CD8^+^ CD45RA/CD27 defined T cell following an 18 hour stimulation with 0.5 μg/ml anti-CD3 and 5 g/ml IL-2. Graphs show the mean ± SEM for 3 donors. (B) Flow cytometry plots and graph showing the expression of phosphorylated AMPK in CD8+ T cell subsets *ex-vivo*. Graph shows the mean ± SEM for 6 donors. (C) Gene expression changes for genes controlling telomerase activity in CD45RA/CD27 defined subsets. The heat map keys show log-fold changes from baseline. **(D) The relative gene expression of CD28 in the N-EMRA and EM-EMRA population.** (E) Graphs showing the production of IL-1β and IL-6 from stimulated CD8^+^ EMRAs following blockade of the p38 MAPK pathway using BIRB796. Graph shows the mean ± SEM for 3 donors. P values were calculated a repeated measures ANOVA with the Tukey correction used for post-hoc testing.
